# Transapical zone 0 thoracic endovascular aortic repair with reversed debranching under extracorporeal membrane oxygenation support

**DOI:** 10.1016/j.xjtc.2025.06.003

**Published:** 2025-06-18

**Authors:** Fumitaka Suzuki, Ryohei Ushioda, Hiroyuki Kamiya

**Affiliations:** Department of Cardiac Surgery, Asahikawa Medical University, Asahikawa, Hokkaido, Japan

**Figure:**
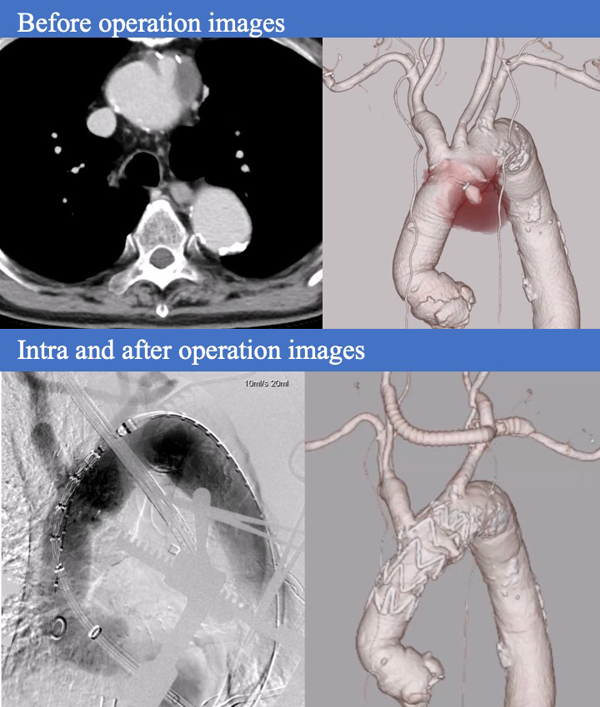
Transapical zone 0 TEVAR using a reversed debranching technique under ECMO.

**Central Message**

This report describes a successful case of transapical zone 0 thoracic endovascular aortic repair using a reversed debranching technique under extracorporeal membrane oxygenation in a frail elderly patient with an ascending aortic pseudoaneurysm.

## Case Presentation

**Figure 1:**
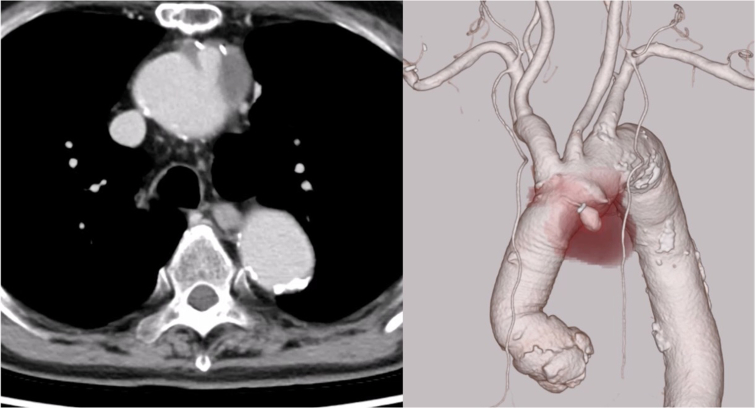
Follow-up computed tomography scan performed 6 years after ascending aortic replacement with a 26-mm J Graft Shield Neo showing a pseudoaneurysm at the anastomotic site on the lesser curvature of the aortic arch, gradually enlarging to 38 mm.

An 80-year-old male had undergone ascending aortic replacement with a 26-mm J graft (J Graft Shield Neo; Japan Lifeline) for acute type A aortic dissection 6 years earlier. Routine postoperative computed tomography angiography (CTA) starting in the second year after surgery revealed an 18-mm anastomotic pseudoaneurysm on the lesser curvature of the aortic arch, opposite the brachiocephalic artery. The lesion gradually enlarged to 38 mm (Figure 1), prompting surgical intervention. Written informed consent was obtained from the patient for publication of this case, and Institutional Review Board approval was not required.

**Figure 2:**
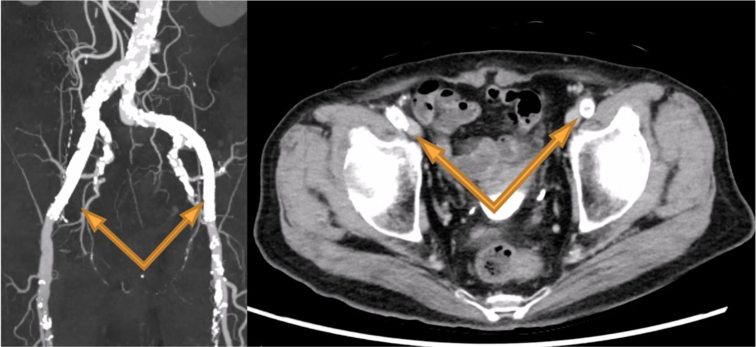
Chronic occlusive arteriosclerosis of the lower limbs. Despite prior bilateral femoral artery stent placements (7 mm × 10 cm; SMART), restenosis developed, limiting transfemoral device access. The *arrow* marks indicate the sites of previous stent placement in the femoral artery interventions, including endarterectomy and stent placement (7 mm × 10 cm; SMART; Cordis), performed due to chronic lower limb arteriosclerosis.

The patient's past medical history included severe chronic obstructive pulmonary disease, lung cancer, and chronic lower limb arteriosclerosis, for which he had previously undergone bilateral femoral artery endarterectomy and endovascular therapy with stent placement (7 mm × 10 cm; SMART; Cordis). Transthoracic echocardiography (TTE) revealed normal aortic valve function, with no evidence of stenosis or regurgitation. Given his frailty and complex vascular anatomy (Figure 2, Video 1), a conventional transfemoral approach was deemed unfeasible; therefore, a transapical approach for thoracic endovascular aortic repair (TEVAR) was selected.

Because the pseudoaneurysm involved zone 0 of the aortic arch, complete debranching of the supra-aortic vessels was required. Bypass grafts were constructed from the left subclavian artery, serving as the inflow source, to both common carotid arteries. We used 8-mm vascular grafts (Propaten; WL Gore) for the arch vessel bypass. To maintain hemodynamic stability and facilitate device manipulation, venoarterial extracorporeal membrane oxygenation (ECMO) was established via the bypass graft and femoral vein. Systemic heparin (100 U/kg) was administered intraoperatively to maintain the activated clotting time between 250 and 3000 seconds.

Through a left thoracotomy at the fifth intercostal space, an 8 Fr sheath was introduced into the left ventricular apex and subsequently exchanged for a 22 Fr balloon sheath. Under rapid ventricular pacing and with the aid of a 0.035-in. stiff guidewire, a stent graft (37 × 37 mm, 90 mm long, Valiant Navion; Medtronic, Santa Rosa, California) was successfully deployed. This graft was selected based on a distal landing zone diameter of 35 mm, providing minimal oversizing for secure sealing. Hemostasis at the left ventricular apical access site was achieved using 4-0 polypropylene mattress sutures reinforced with 2 large felt pledgets. After confirming hemodynamic stability, the patient was smoothly weaned from ECMO support in the operating room.

**Figure 3:**
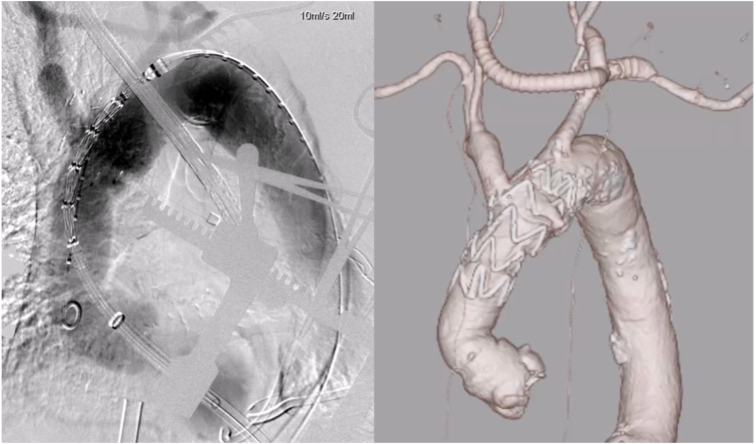
Because zone 0 TEVAR was indicated, a bypass from the left subclavian artery to both common carotid arteries was created. Extracorporeal membrane oxygenation was established via the bypass and femoral vein. A stent graft (37 mm × 37 mm, 90 mm; Valiant Navion) was deployed via the transapical approach under rapid pacing. Postoperative imaging confirmed successful exclusion of the pseudoaneurysm without complications.

Postoperative CTA on day 7 confirmed complete exclusion of the pseudoaneurysm, with no evidence of endoleak (Figure 3). Therefore, no additional embolization or coiling of the brachiocephalic artery was performed. Postoperative TTE demonstrated preserved aortic valve function. The patient experienced an uneventful postoperative course and was discharged 2 weeks after surgery. Unfortunately, he died from lung cancer at 18 months postoperatively.

## Discussion

Anastomotic pseudoaneurysms are serious late complications of aortic surgery, associated with a high risk of rupture and poor outcomes when managed conservatively.^1^ For frail or high-risk patients, debranching TEVAR offers a less invasive alternative to conventional total arch replacement.

Previous reports on zone 0 TEVAR for anastomotic aneurysms have described techniques combining full supra-aortic debranching with chimney grafts for brachiocephalic artery revascularization.^2^ In our case, the left subclavian artery was selected as the inflow source to minimize the risk of gutter endoleaks and reduce the likelihood of future reintervention.

While the transfemoral route is the standard access for TEVAR, alternative approaches—such as direct access via the common iliac artery, abdominal aorta, ascending aorta, common carotid artery, or subclavian artery—have been reported in cases with difficult femoral access.^3^ In the present case, a transapical approach was selected because it was a reoperation requiring total debranching TEVAR in the shaggy aorta.

The adjunctive use of ECMO has been proposed to improve procedural safety during complex aortic repairs. ECMO not only ensures adequate end-organ perfusion, but also minimizes hemodynamic fluctuations and the risk of cerebral ischemia.^4^ When combined with rapid pacing, it enables precise stent deployment and controlled cardiac output during critical procedural steps.^5^

This case demonstrates the feasibility and safety of a transapical zone 0 TEVAR with reversed debranching under ECMO support. This strategy may serve as a valuable option for managing elderly and frail patients with complex aortic pathologies and limited vascular access.

## Conflict of Interest Statement

The authors reported no conflicts of interest.

The *Journal* policy requires editors and reviewers to disclose conflicts of interest and to decline handling or reviewing manuscripts for which they may have a conflict of interest. The editors and reviewers of this article have no conflicts of interest.

## Supplementary Data

Video 1 Preoperative contrast-enhanced computed tomography angiography showing a 38-mm anastomotic pseudoaneurysm on the lesser curvature of the aortic arch, a severely shaggy aorta, and significant peripheral vascular disease. Video available at: https://www.jtcvs.org/article/S2666-2507(25)00252-4/fulltext.
