# Supplementary figures and images for: Transapical zone 0 thoracic endovascular aortic repair with reversed debranching under extracorporeal membrane oxygenation support
Source: JTCVS Tech. 2025 Jun 18;33:31–4. doi: 10.1016/j.xjtc.2025.06.003 (PMC12529721; doi:10.1016/j.xjtc.2025.06.003)

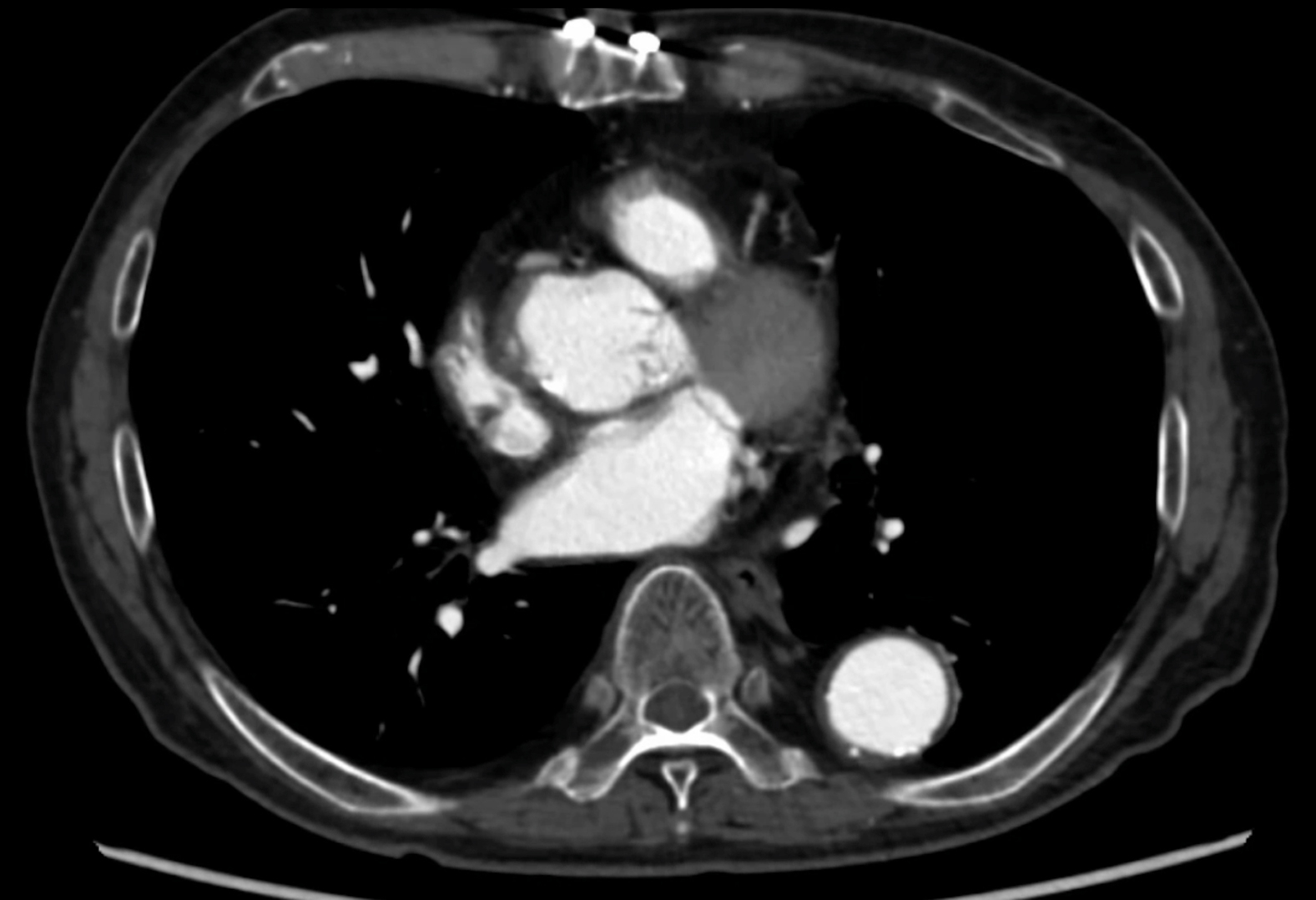

Supplement: Video 1 — Preoperative contrast-enhanced computed tomography angiography showing a 38-mm anastomotic pseudoaneurysm on the lesser curvature of the aortic arch, a severely shaggy aorta, and significant peripheral vascular disease. Video available at: https://www.jtcvs.org/article/S2666-2507(25)00252-4/fulltext. [file fx2.jpg]
